# Supplementary material for: Structural interplay between DNA-shape protein recognition and supercoiling: The case of IHF
Source: Comput Struct Biotechnol J. 2022 Sep 19;20:5264–74. doi: 10.1016/j.csbj.2022.09.020 (PMC9519438; doi:10.1016/j.csbj.2022.09.020)
Supplement: Supplementary file 1 [file mmc1.pdf]

Structural interplay between DNA-shape protein recognition and supercoiling: the case of IHF

George D. Watson[1], Elliot W. Chan[1], Mark C. Leake[1,2], Agnes Noy[1*]

[1] Department of Physics, University of York, York YO10 5DD, United Kingdom.

[2] Department of Biology, University of York, York YO10 5DD, United Kingdom.

[*] Correspondence to: agnes.noy@york.ac.uk.

Supplementary Information

DNA sequence

The DNA sequence used for these simulations is a 336 bp piece of covalently closed circular DNA. The IHF consensus sequence occurs once in this construct (underlined), where the most conserved bases are highlighted in bold.

[1]GAACGAGAAA [11]CGTAAAATGA [21]TATAAA**TATC** [31]**AA**TATA**TTA**A [41]ATTAGATTTT [51]GCATAAAAAA

[61]CAGACTACAT [71]AATACTGTAA [81]AAACCATCTG [91]CGGTGATAAA [101]TTATCTCTGG [111]CGGTGTTGAC

[121]ATAAATACCA [131]CTGGCGGTGA [141]TAGCTGAGGC [151]ATCTTTCTAC [161]ATGAAAGTGT [171]CGGAACTCG

[181]TACATAGGCT [191]TCCGGCTGAT [201]AGTAGTCGTA [211]GTAGGAAACG [221]AAATATTCGA [231]CCGCGTTTT

[241]CGGGAAGTAT [251]CACCGCCAGA [261]GGTAAAATAG [271]TCAACACGCA [281]CGGTGTTAGA [291]TATTTATCC

[301]TTGCGGTGAT [311]ATCGCTCAAC [321]TTAGTATAAA [331]AAAGCT

Supplementary Figures

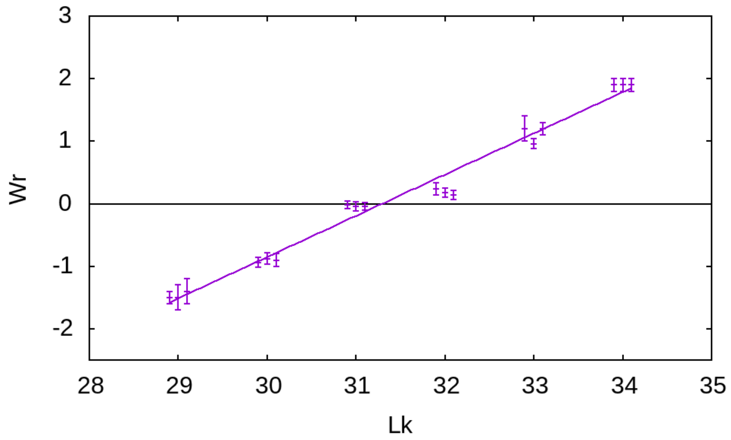

Figure S1. Linear fit of writhe against linking number. The value of Lk corresponding to torsionally relaxed DNA (Lk_0) was identified by plotting the writhe (calculated using WrLINE) against the number of turns in the minicircle for each of a series of simulations of bare DNA.

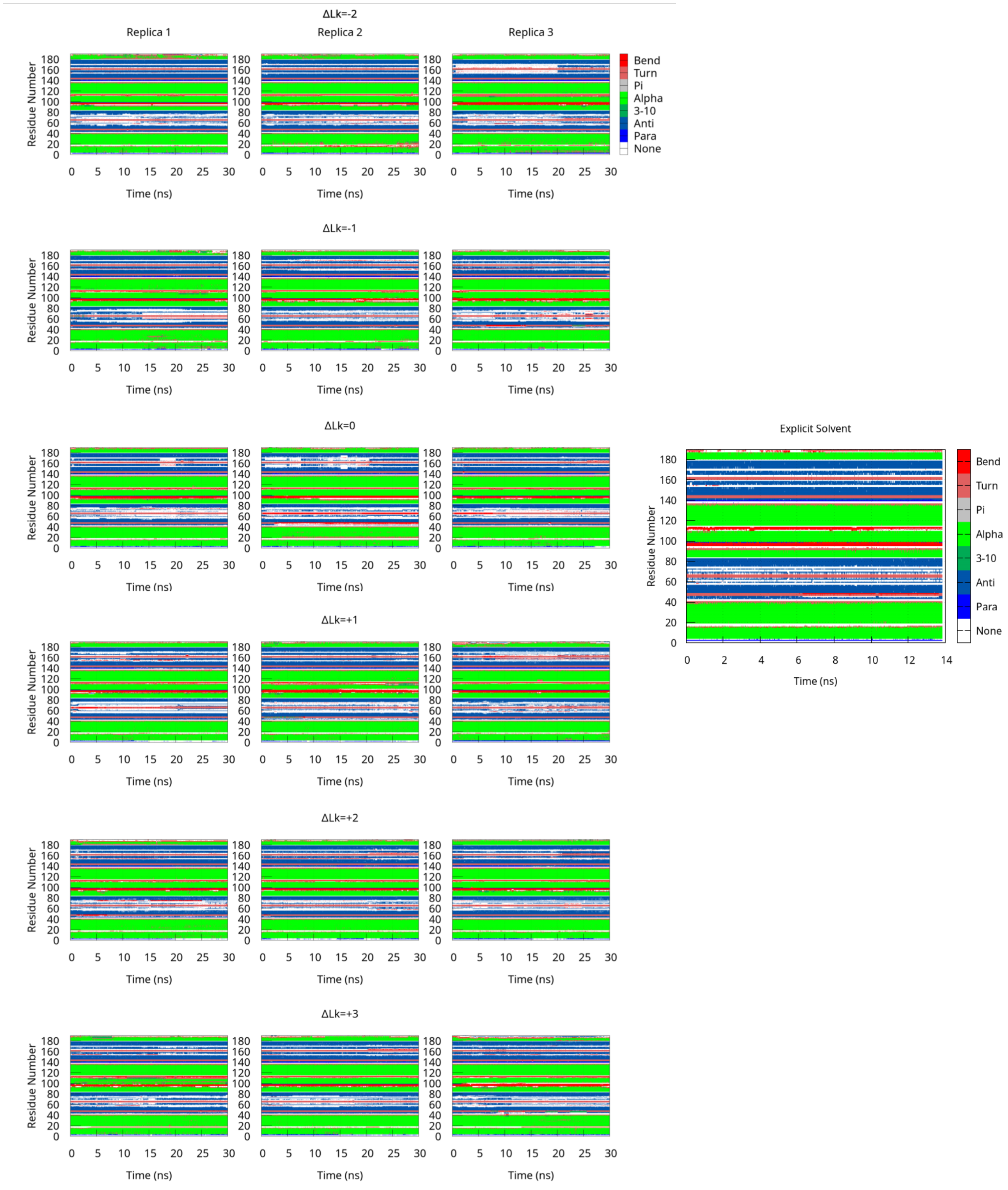

Figure S2. Designation of the secondary structure of IHF amino acids using the DSSP algorithm along all replicas in implicit solvent and a simulation in explicit solvent extracted from our previous study (ref. 10). Secondary structure is mostly maintained during our simulations (either in implicit or in explicit solvent) with the exception of some of the antiparallel beta sheets placed in IHF's extended arms. This part of the protein folding has been described as highly flexible according to several NMR and crystallographic structures (like PDBs 1HUE, 1WTU, 5OGU, 1EXE, 6OAJ) remaining often unsolved.

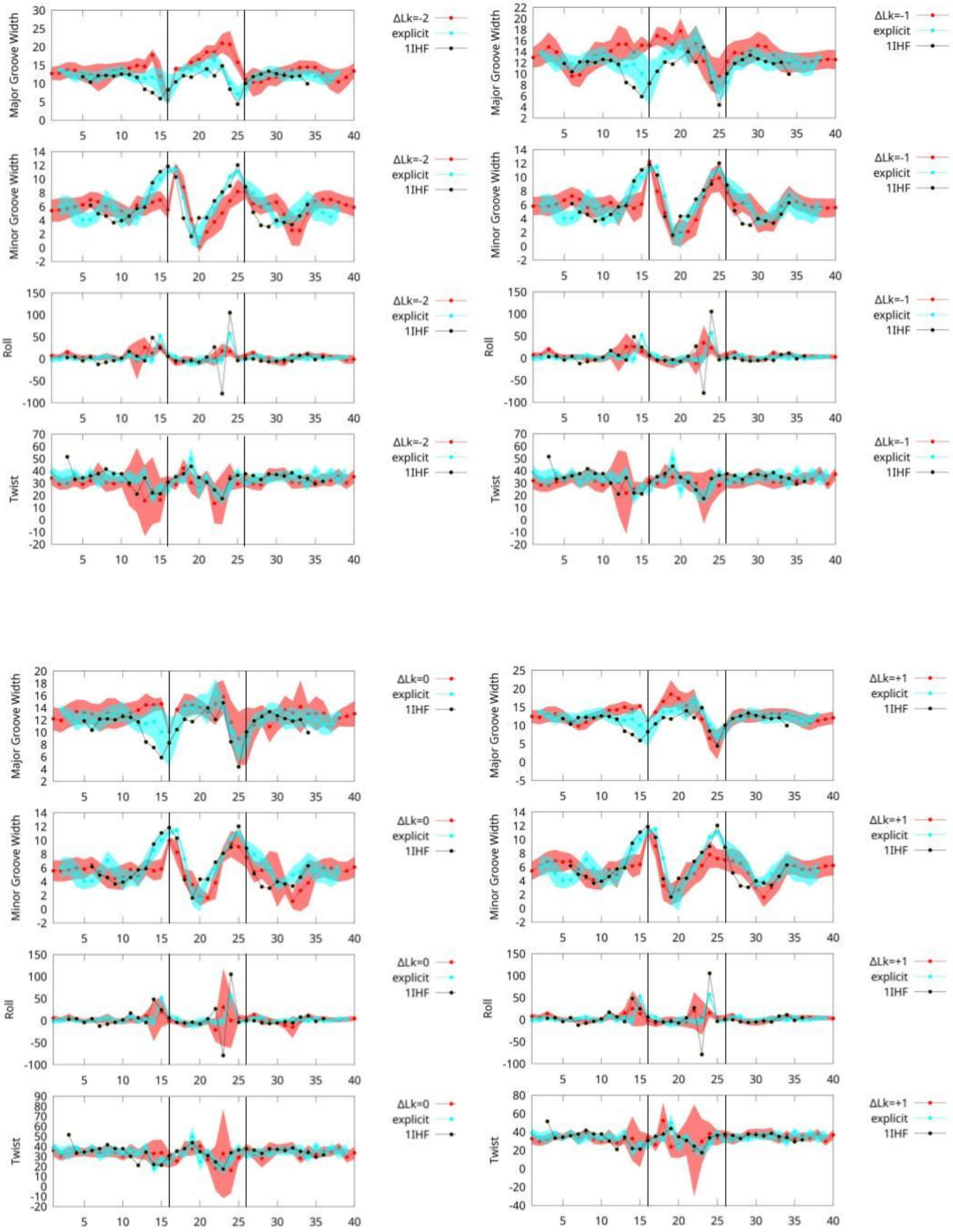
Major Groove Width
Minor Groove Width
Roll
Twist
ΔLk=-2
ΔLk=-1
ΔLk=0
ΔLk=+1
explicit
1IHF

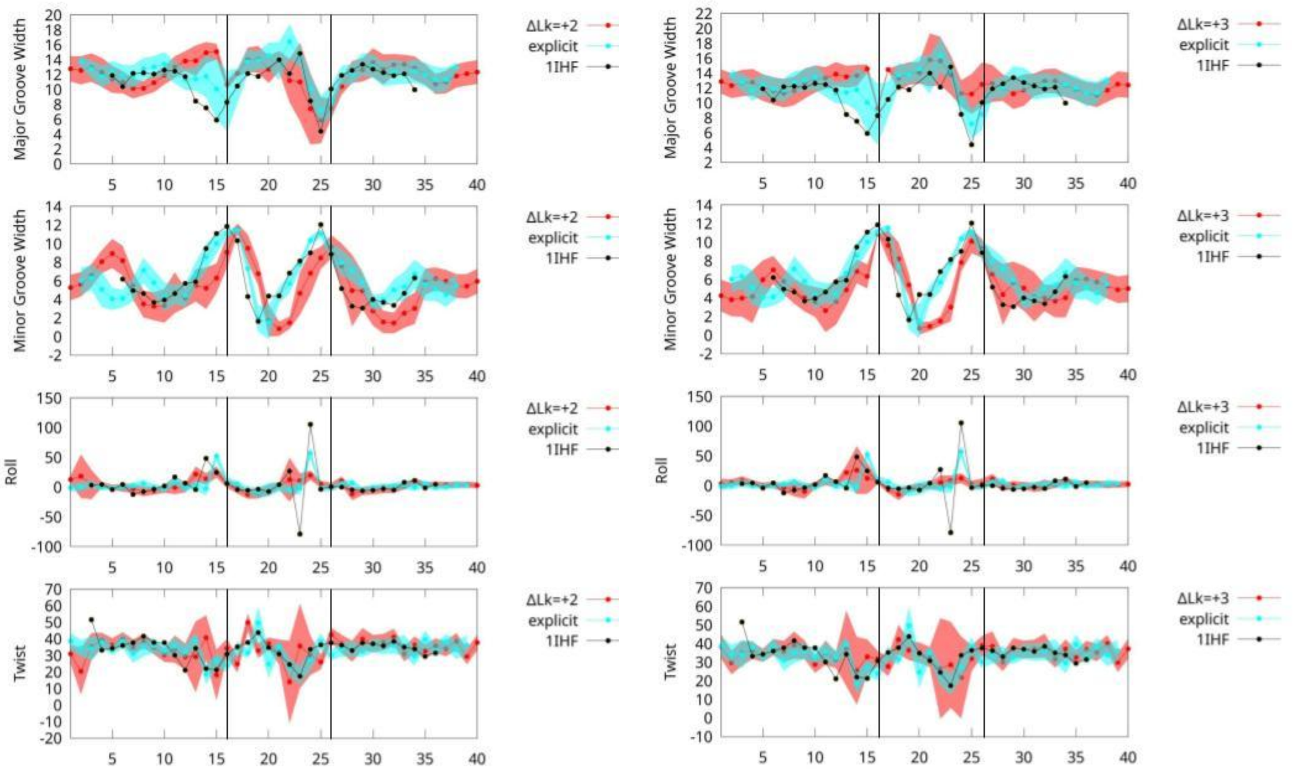

Figure S3. Minor and major groove widths together with roll and twist at the dinucleotide level along IHF binding site for all six implicitly solvated topoisomers compared with the crystal PDB 1IHF and an explicitly solvated simulation of IHF bound to linear DNA (10). Only the central 11-bp between the two intercalation sites (separated by vertical lines) was kept from the experimental structure when embedding IHF to circular DNA. We observed best agreement on the relaxed topoisomer, as the X-ray structure and the simulation in explicit solvent were done on linear/relaxed DNA. The last 10ns of the three replicas from each topoisomer were concatenated in a single trajectory in order to obtain the reported average values and standard deviations (given as shade areas)

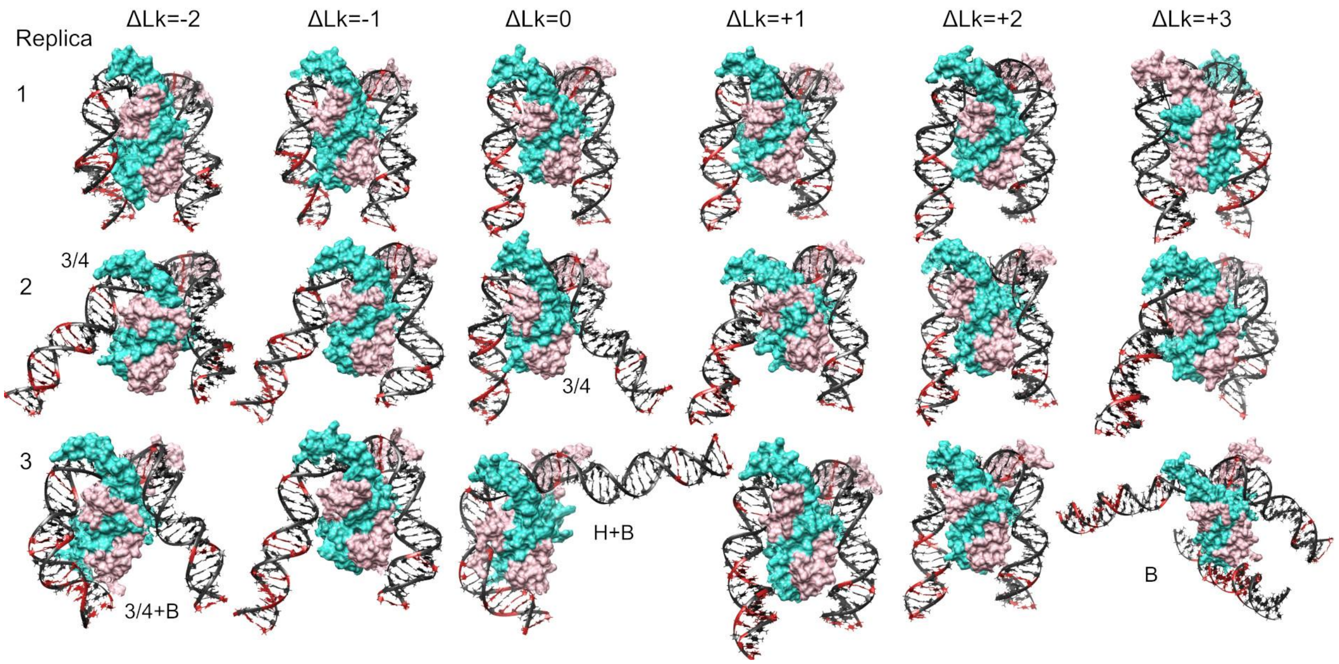

Figure S4. Supercoiling dependence of DNA structure when bound to IHF observed for all replicas. The only few CG bp in the binding site are highlighted in red and serve as rulers to compare DNA orientation relative to IHF sides. The color scheme is the same as in Figure 5.

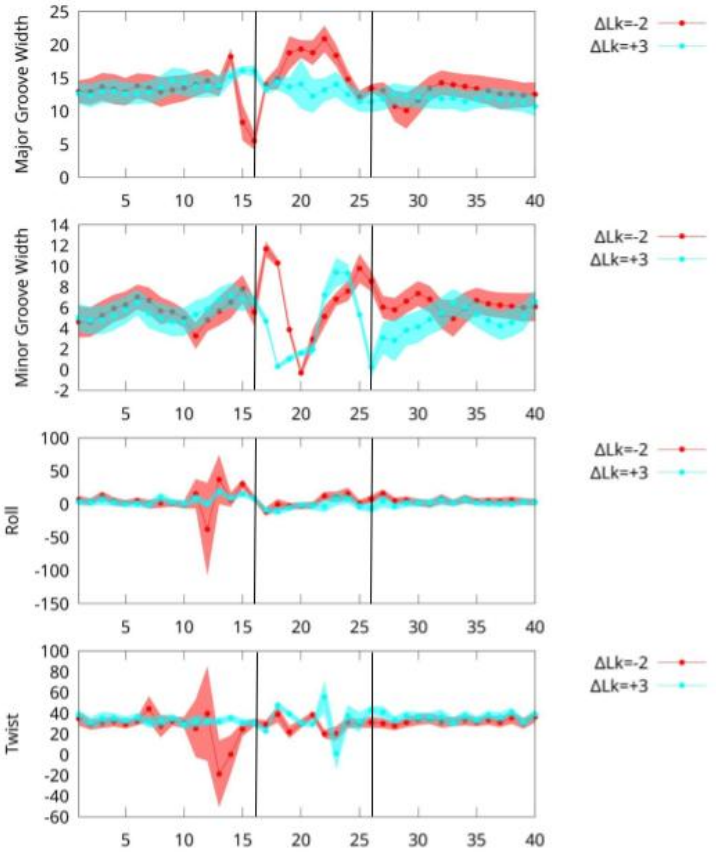

Figure S5. Minor and major groove widths together with roll and twist at the dinucleotide level along IHF binding site for replica 1 of the most negatively and positively supercoiled topoisomers. We compare these replicas because they are the ones where the DNA is most tightly packed around the protein (see Figure S5) and global twist is most different at the binding site (see Figure 4D). We observe large differences on grooves widths; and modest but consistent differences on bp-step twists that, when accumulated, generate significant restrained torsion as shown in Figure 4. The reported values are averages over the last 10 ns of the simulations and the corresponding standard deviations are given as shade areas.

Supplementary Movies

Supplementary Movie 1. First replica of topoisomer ΔLk=-2 where the IHF-DNA complex adopts the fully wrapped state.

Supplementary Movie 2. Second replica of topoisomer ΔLk=-2 where the IHF-DNA complex adopts the three-quarters state.

Supplementary Movie 3. Third replica of topoisomer ΔLk=-2 where the IHF-DNA complex adopts the three-quarters + bottom .

Supplementary Movie 4. First replica of topoisomer ΔLk=-1 where the IHF-DNA complex adopts the fully wrapped state.

Supplementary Movie 5. First replica of topoisomer ΔLk=0 where the IHF-DNA complex transitions from the three-quarters to the fully wrapped state.

Supplementary Movie 6. Second replica of topoisomer ΔLk=0 where the IHF-DNA complex adopts the three-quarters state.

Supplementary Movie 7. Third replica of topoisomer ΔLk=0 where the IHF-DNA complex adopts the half-wrapped + bottom state.

Supplementary Movie 8. First replica of topoisomer ΔLk=+1 where the IHF-DNA complex adopts the fully wrapped state.

Supplementary Movie 9. First replica of topoisomer ΔLk=+2 where the IHF-DNA complex adopts the fully wrapped state.

Supplementary Movie 10. First replica of topoisomer ΔLk=+3 where the IHF-DNA complex adopts the fully wrapped state.

Supplementary Movie 11. Second replica of topoisomer ΔLk=+3 where the IHF-DNA complex adopts the fully wrapped state in a compact trefoil conformation.

Supplementary Movie 12. Third replica of topoisomer ΔLk=+3 where IHF mediates a bridge between two distal DNA sites.
